# E-TBNet: Light Deep Neural Network for Automatic Detection of Tuberculosis with X-ray DR Imaging

**DOI:** 10.3390/s22030821

**Published:** 2022-01-21

**Authors:** Le An, Kexin Peng, Xing Yang, Pan Huang, Yan Luo, Peng Feng, Biao Wei

**Affiliations:** 1 College of Computer Science and Cyber Security, Chengdu University of Technology, Chengdu 610059, China; anle@stu.cdut.edu.cn (L.A.); 2020020863@stu.cdut.edu.cn (X.Y.); 2 The Key Laboratory of Optoelectronic Technology and Systems, Ministry of Education, Chongqing University, Chongqing 400044, China; panhuang@cqu.edu.cn (P.H.); 20152572@cqu.edu.cn (Y.L.); coe-fp@cqu.edu.cn (P.F.); weibiao@cqu.edu.cn (B.W.)

**Keywords:** tuberculosis detection, chest X-ray images, neural network, embedded device

## Abstract

Currently, the tuberculosis (TB) detection model based on chest X-ray images has the problem of excessive reliance on hardware computing resources, high equipment performance requirements, and being harder to deploy in low-cost personal computer and embedded devices. An efficient tuberculosis detection model is proposed to achieve accurate, efficient, and stable tuberculosis screening on devices with lower hardware levels. Due to the particularity of the chest X-ray images of TB patients, there are fewer labeled data, and the deep neural network model is difficult to fully train. We first analyzed the data distribution characteristics of two public TB datasets, and found that the two-stage tuberculosis identification (first divide, then classify) is insufficient. Secondly, according to the particularity of the detection image(s), the basic residual module was optimized and improved, and this is regarded as a crucial component of this article’s network. Finally, an efficient attention mechanism was introduced, which was used to fuse the channel features. The network architecture was optimally designed and adjusted according to the correct and sufficient experimental content. In order to evaluate the performance of the network, it was compared with other lightweight networks under personal computer and Jetson Xavier embedded devices. The experimental results show that the recall rate and accuracy of the E-TBNet proposed in this paper are better than those of classic lightweight networks such as SqueezeNet and ShuffleNet, and it also has a shorter reasoning time. E-TBNet will be more advantageous to deploy on equipment with low levels of hardware.

## 1. Introduction

According to the “Global Tuberculosis Report 2020” issued by the World Health Organization (WHO), the number of new tuberculosis patients in China in 2019 was approximately 833,000, ranking third in the world [1]. Due to the lack of experienced physicians or related diagnostic equipment in China’s economically underdeveloped remote areas, the prevention and treatment of tuberculosis in primary hospitals is difficult. The use of “Internet +” technology can improve the level of screening of tuberculosis patients in primary hospitals, which is also an important part of effective prevention and treatment of tuberculosis. In underdeveloped areas, digital radiography (DR) technology is widely used in tuberculosis screening, and chest radiographs are examined by experienced physicians for the diagnosis of TB. However, physicians in primary hospitals have less experience in reading such radiographs, and the imaging quality of the DR equipment is not good. The above reasons can easily lead to misdiagnosis and/or missed diagnosis of TB. As a result, computer-assisted system technology came into being to assist doctors in identifying disease. With the widespread application of deep learning technology in the field of medical image processing, the accuracy of convolutional neural networks (CNNs) in the detection of tuberculosis has also been continuously improved.

In the field of deep learning, Lakhani et al. [2] first explored the ability of deep convolutional neural networks to detect pulmonary tuberculosis via chest X-ray images. The experimental results show that the AUC (area under the curve) of the best performing model in the detection of tuberculosis was 0.99; the disadvantage of this model is that it has tens of millions of parameters, higher computational complexity, and is difficult to configure on much cheaper and less powerful hardware.

The accuracy of CNNs is closely related to the quality of the image dataset, and a large number of high-quality training samples are more capable of producing excellent models. In terms of image quality, Munadi et al. [3] proved through experiments that using unsharp masking (UM) image enhancement algorithms or high-frequency emphasis filtering (HEF) on chest X-ray images of tuberculosis patients can effectively improve the judgment ability of CNNs. In terms of image quantity, Liu et al. [4] further promoted the development of computer-aided tuberculosis diagnosis (CTD), and constructed a large-scale gold-standard tuberculosis dataset with dual labels for classification and location, which can be used for TB research.

Due to the 2D characteristics of the chest X-ray images, the overlap or occlusion of multiple tissues and organs seriously affects the recognition of neural networks. In order to increase CNNs’ attention to the lung area of the chest radiographs, Rahman et al. [5] used an image segmentation network to segment the lung region, then sent the segmented image to the CNNs. A variety of network experiments have proven that image segmentation can significantly improve classification accuracy, but the two-stage detection algorithm has problems, such as heavy model weight and longer reasoning time.

In addition to improving the image quality to enhance CNNs’ detection of tuberculosis disease, researchers have also conducted many explorations of the model structure. Henghao et al. [6] used the transfer learning approach to use the pneumonia deep network detection model to train the feature extraction subnetwork of chest radiographs, and proposed a deep learning detection algorithm based on focal loss—Tuberculosis Neural Net (TBNN); the AUC of the model’s detection is 0.91. Rajaraman et al. [7] used different CNN structures to transfer and learn the same modal pneumonia images, then used ensemble learning to further improve the model’s accuracy. Stacking ensemble learning demonstrated better performance in terms of performance metrics (accuracy (0.941), AUC (0.995)). Although transfer learning can solve the training problem of CNNs on small-scale dataset, other researchers have designed more complex and heavy neural network structures, which will be a huge test for devices with limited computing power and storage power; the reasoning process of these models faces the problems of longer execution time and difficulty in deployment.

**Figure 1:**
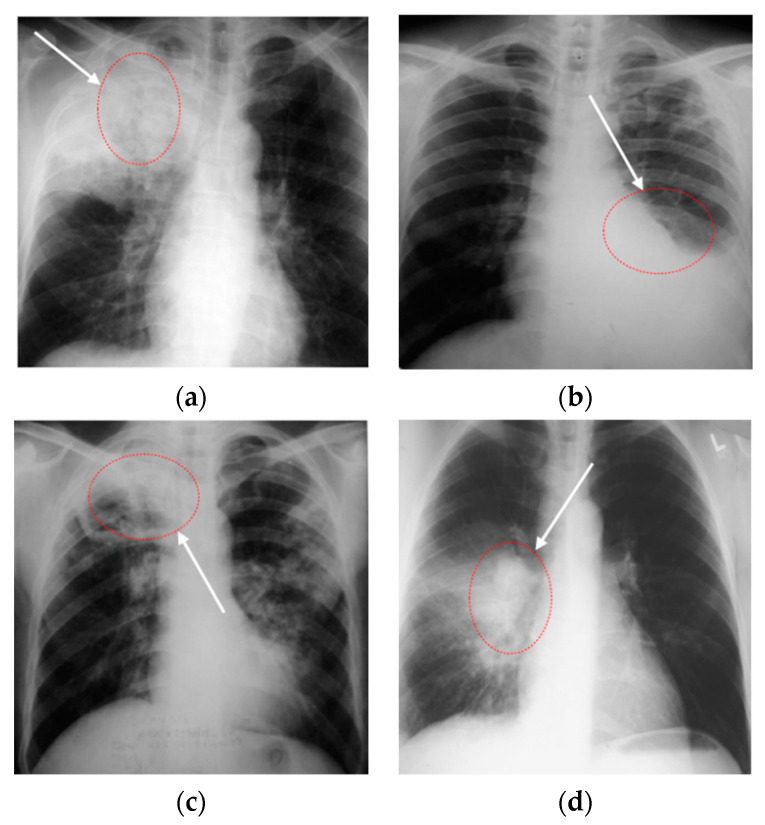
Chest X-ray images of individual tuberculosis patients: (**a**) consolidation of the upper lobe of the right lung with cavities; (**b**) tuberculous exudative pleurisy; (**c**) secondary tuberculosis of right upper lung; (**d**) consolidation of right hilar with enlarged lymph nodes.

Through the above research, analysis, and related experiments, it was found that, firstly, the effect of the lung segmentation algorithm on chest X-ray images is affected by the different imaging manifestations of tuberculosis. Figure 1 shows four X-ray images of TB patients, and the marked areas in Figure 1a,b are the lesions. The segmentation of lung parenchyma leads to the omission of some focal points. In Figure 1c,d, the ribs and lesions in the marked area partially coincide. If rib suppression or lung parenchyma segmentation is performed, the focus will be hidden. After the chest X-ray images of the above four types of tuberculosis are segmented, some of the lesions are missing, resulting in poor performance in CNN classification. Secondly, the classic classification deep CNNs (VGGNet [8], DenseNet [9], etc.) and variant networks similar to them have excellent performance, but they are difficult to deploy on devices with low hardware, due to the amount of model parameters, computational complexity, and weights.

Based on the above viewpoints, the specific work of this article is mainly carried out in the following three parts:

(1) In the first part, we first analyzed the distribution characteristics of the two publicly available small-scale chest X-ray image datasets of TB, then designed dataset fusion rules and, finally, specifically built the backbone of an efficient TB detection model for PCs or embedded devices with less powerful hardware;
(2) In the second part, we first introduced an efficient channel attention (ECA) mechanism and residual module, then improved them and added them to the network structure, and finally built the detailed network architecture through multiple experiments. While compressing model calculations and reducing model parameters, this keeps the accuracy of the model in line with the expected level, so that it has a visible advantage in clinical practice;
(3) In the third part, the network proposed in this paper was compared to classical lightweight networks through quantitative indicators such as Sensitivity, Specificity, Accuracy, Precision, Times, etc., and then the model’s reasoning efficiency was evaluated in two different hardware environments.

## 2. Materials and Methods

### 2.1. Deep Residual Network

Simple accumulation of a deep neural network structure causes network degradation. At this time, the shallow network can obtain better training than the deep network, because the loss of the deep network training process will decrease and then tend to be saturated. When the number of network layers is increased, the loss will increase. To solve this problem, a deep residual network (ResNet) [10] was proposed, which can effectively eliminate the gradient dispersion or gradient explosion caused by the increase in the number of model layers.

**Figure 2:**
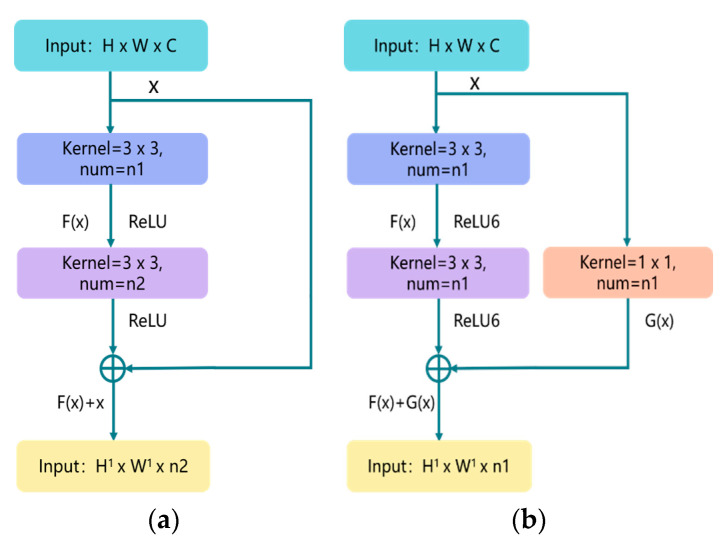
The residual block structure: (**a**) the traditional residual block structure; (**b**) the improved residual block structure. H, W, and C represent the height, width, and channel of the image, respectively; num represents the number of convolution kernels.

**Figure 3:**
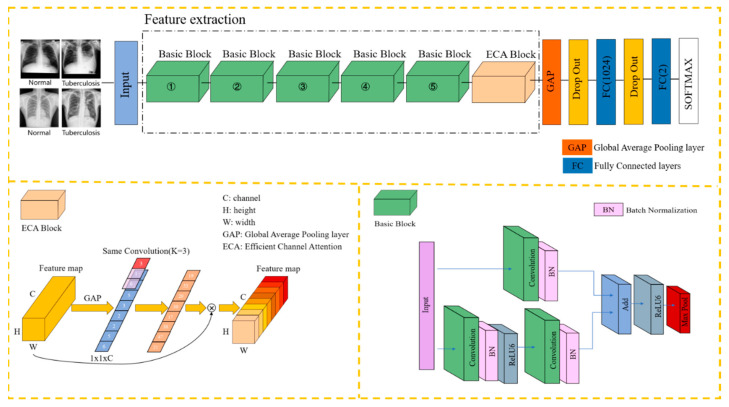
The framework of the proposed method of tuberculosis detection is based on chest X-ray images, consisting of two parts: the ECA block and the basic block. We found through a large number of ablation experiments that inserting the ECA block in this article after position 5 is the best. The model shown in Figure 3 is the optimal model.

Figure 2a illustrates the overview of residual structure, which consists of two parts: the identity shortcut connection x, and the residual mapping F(x). x and F(x) + x represent the input and output of the residual module, respectively, while channel represents the output channel of the residual module. For each residual structure we use a stack of 2 layers. The residual module used in this article is shown in Figure 2b; the purpose of adding 1 × 1 convolution in the shortcut connection direction is to cleverly control the dimensional change of the output feature map. The dimensions (w × h) of F(x) and G(x) must be equal; otherwise, the stride of the 1 × 1 convolution layer can be changed to adjust the dimensions. At the same time, we use the ReLU6 activation function to adjust the maximum value of the ReLU output to 6, in an effort to prevent loss of accuracy when running on low-precision mobile devices (float18/int8). The details of the basic block are shown in Figure 3 (Basic Block).

### 2.2. Attention Mechanism

Today, channel attention mechanisms in computer vision offer great potential for improving the performance of CNNs. In 2017, squeeze-and-excitation networks (SENets) first proposed a flexible and efficient channel attention mechanism [11], the principle of which is that the global average pooling operation is used to compress the feature map into a real number, and then the real number is input into a network composed of two fully connected layers; the network’s output is the weight of the feature map in the channel axis, and the two FC layers are designed to capture nonlinear cross-channel interaction. In 2020, researchers empirically showed that appropriate cross-channel interaction can preserve performance while significantly decreasing model complexity; therefore, they proposed an efficient channel attention (ECA) module for model complexity and computational burden brought by SENet [12]. As illustrated in Figure 3 (ECA Block), ECA uses an adaptively adjustable one-dimensional convolution kernel (kernel size = 3) to replace the original fully connected layer, and it effectively fuses the information of adjacent channels. The calculation process of the self-adaptive k is shown in Equation (1).

(1) $$k=\Psi \left(C\right)=│\frac{\log\left(C\right)}{\gamma }+\frac{b}{\gamma }│$$

where *k* is the kernel size of the adaptive convolutional layer, and *C* is the number of channels of the input feature map. In the original paper, *b* and *γ* were set to 1 and 2, respectively. In a brief conclusion, this paper draws on the ECA-Net, which effectively improves the performance while adding only a few parameters.

### 2.3. Network Structure

Integrating the content of the previous section with the E-TBNet classification network architecture proposed in this paper, Figure 3 illustrates the overview of our E-TBNet.

As shown in Figure 3, the feature extraction part of the network is composed of 5 groups of basic blocks with similar structures. The basic blocks include residual connections and direct connections. The residual connections are composed of two 3 × 3 convolutional layers in order to extract features from the input image. The number of convolution kernels in each layer remains the same. The numbers of convolution kernels for the 5 groups of basic blocks are 16, 32, 48, 64, and 128, respectively.

The direct connection contains only one 1 × 1 convolutional layer; its role is to adjust the size (w × h) and the channel of input image. When the size of the input image changes after the residual connection, the direct connection can adjust the size of feature map by changing the 1 × 1 convolution layer’s stride.

After the convolution operation in the basic blocks, we perform the ReLU6 activation function to increase the nonlinearity of the neural network; the output value of ReLU6 is limited to 6 at most. If there is no restriction, low-precision embedded devices cannot accurately trace large values, which will lead to a decrease in model accuracy. The stride of the first basic block is set to 2, and the image size is changed by downsampling. Because the first layer of convolution extracts more basic information, such as the edges and textures of the target, the downsampling operation does not reduce the model accuracy, and can reduce the computational complexity. The strides of the next four basic blocks are set to 1, and the size of the input remains same. At this time, according to the receptive field calculation formula in Equation (2), it can be seen that two 3 × 3 convolution kernels and one 5 × 5 convolution kernel have the same receptive field, while a larger receptive field will capture semantic information in a larger neighborhood.

(2) $$RF_{i}=\left(RF_{i+1}-1\right)\times stride_{i}+Ksize_{i}$$

In Equation (2), *RF_i_* and *RF_i_*_+1_ represent the receptive field of the *i*th and *i*th + 1 convolutional layers, respectively, while *stride_i_* and *Ksize_i_* represent the stride and the size of the convolution kernel of the *i*th convolutional layer, respectively.

We perform the max pooling operation directly by each basic block, obtaining the maximum value of the pixels in the neighborhood to remove redundant information and retain decision-making information. The image appearance of tuberculosis lesions is irregular—mostly dots, clouds, and bars. Multiple pooling operations can retain the salient features extracted in the filters, while reducing the size of the feature map and increasing the calculation speed. A basic block and a pooling layer are connected in series to form the feature extraction part of the network in this paper.

The ECA blocks in Section 2.2 produce neither extra parameters nor computation complexity; this is extremely attractive in practice. In order to further improve the robustness of the model, the ECA block is inserted after the basic block. The feature information is compressed to one dimension through the global average pooling operation, and each feature map is mapped onto a single value. After the average max pooling layer, the two fully connected layers complete the classification. Considering that the sample data are fewer, in order to alleviate the overfitting of the model, the dropout layer is added after the global average pooling layer and the first fully connected layer, and the rate is set to 0.5. In each training batch, 1/2 of the neuron nodes are randomly inactivated, in an effort to prevent the model from relying too much on local features and improve its generalization ability. Table 1 shows the hyperparameter settings for model training.

### 2.4. Performance Matrix

In tuberculosis detection tasks, we evaluate the different models through quantitative indicators such as accuracy, sensitivity/recall, specificity, precision/PPV (positive predictive value), NPV (negative predictive value), F1-score, −LR (negative likelihood ratio), and +LR (positive likelihood ratio).

(1) *Accuracy*: We use *accuracy* to evaluate the model’s ability to predict correctly for all samples; this does not consider whether the predicted sample is positive or negative; the formula is shown in (3):

(3) $$Accuracy=\frac{TP+TN}{TP+TN+FP+FN}$$

(2) *Sensitivity*/*Recall*: We use *recall* to analyze what proportion of the samples that are true positives are predicted correctly; recall is also called sensitivity; the formula is shown in (4):

(4) $$Sensitivity/Recall=\frac{TP}{FN+TP}$$

(3) *Specificity*: *Specificity* is relative to sensitivity (*recall*), which represents the ability of the model to correctly predict negative examples from all negative samples; the formula is shown in (5):

(5) $$Specificity=\frac{TN}{FP+TN}$$

(4) *Precision*/*PPV*: In order to analyze the proportion of samples whose predictions are positive, we use *precision*/*PPV* for comparison; the formula is shown in (6):

(6) $$Precision/PPV=\frac{TP}{TP+FP}$$

(5) *NPV*: We use *NPV* to analyze what proportion of the samples that are true negatives are predicted correctly; the formula is shown in (7):

(7) $$NPV\left(negative\;predictive\;value\right)=\frac{TN}{TN+FN}$$

(6) *F*1-*score*: *F*1-*score* is also called balanced *F-score*, and is defined as the harmonic average of *precision* and *recall*. Using *F*1-*score*, we can comprehensively compare *precision* and *recall*; the formula is shown in (8):

(8) $$F1\;Score=\frac{2\times Precision\times Recall}{Precision+Recall}=\frac{2\times TP}{2TP+FN+FP}$$

(7) −*LR*: Likelihood ratio is an indicator that reflects authenticity; in both *sensitivity* and *specificity*, the −*LR* is the ratio of the true positive rate to the false positive rate of the screening results; the formula is shown in (9). +*LR* is the ratio of the false negative rate to the true negative rate of the screening results; the formula is shown in (10):

(9) $$-LR=\frac{1-Sensitivity}{Specificity}=\frac{FN\left(TN+FP\right)}{TN\left(TP+FN\right)}$$

(10) $$+LR=\frac{Sensitivity}{1-Specificity}=\frac{TP\left(FP+TN\right)}{FP\left(TP+FN\right)}$$

Here, true positive (*TP*), true negative (*TN*), false positive (*FP*), and false negative (*FN*) represent the number of tuberculosis images identified as tuberculosis, the number of normal images identified as normal, the number of normal images identified as tuberculosis, and the number of tuberculosis images identified as normal, respectively.

## 3. Results and Discussion

### 3.1. Experimental Conditions

In this paper, a PC and an NVIDIA Jetson AGX Xavier were selected as the network testing experimental platforms. The detailed hardware parameters of the platforms are shown in Table 2. The GPU of the PC is an NVIDIA TITAN V; in terms of performance, the TITAN V has 15 TFLOPS (floating-point operations per second; 1 TFLOPS: 1 trillion floating-point operations) for single-precision floating points, and 7.5 TFLOPS for double-precision floating points; its memory architecture and processor links have achieved great innovations, and have performed well in the field of scientific computing, providing solutions for AI computing and supercomputing.

**Figure 4:**
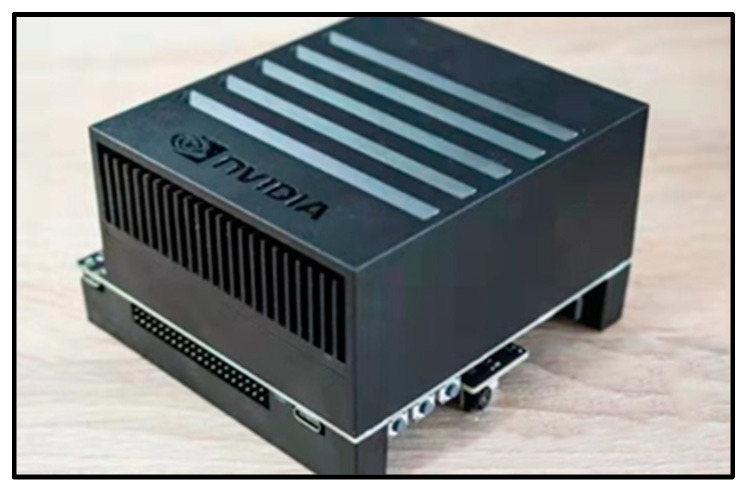
Real image of the Jetson AGX Xavier device.

As shown in Figure 4, the NVIDIA Jetson AGX Xavier platform is the latest version of all Jetson platforms released by NVIDIA, and it uses a Xavier processor. When the platform is running, its power consumption is maintained between 10 W and 30 W, so it is suitable for manufacturing, logistics, smart cities, and smart medical applications [13,14,15,16].

### 3.2. Datasets and Description

**Figure 5:**
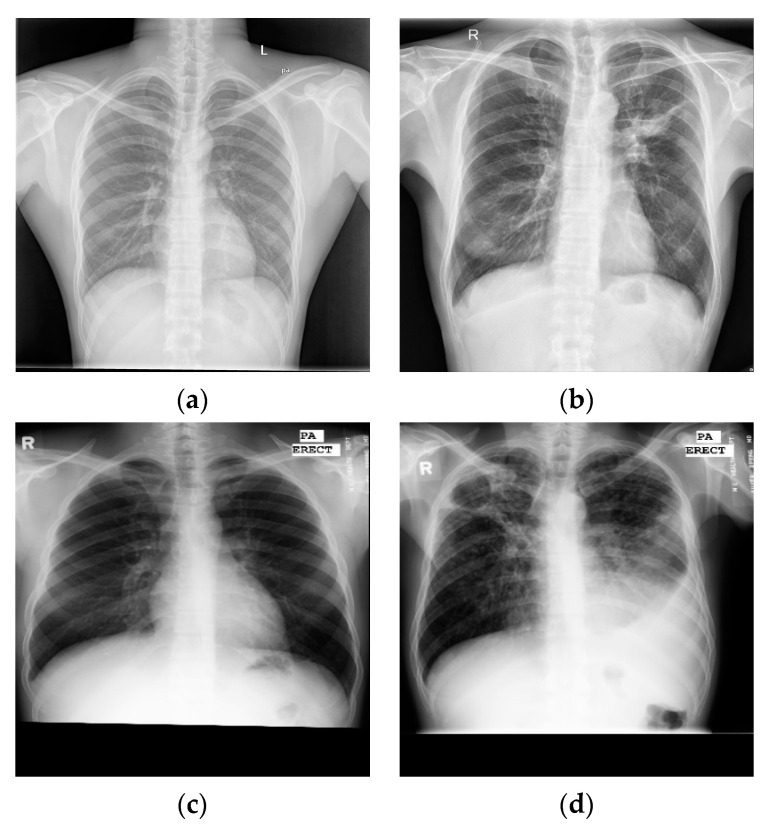
Examples of chest X-ray images from CHN datasets: (**a**) normal chest X-ray; (**b**) tuberculosis chest X-ray. Examples of chest X-ray images from MC datasets: (**c**) normal chest X-ray; (**d**) tuberculosis chest X-ray.

Here, this article uses the posterior anterior chest X-ray image datasets publicly released by the National Library of Medicine (NLM) [17]—the Montgomery and Shenzhen datasets—as shown in Figure 5, where the labels of each image are marked by experienced radiologists.

(1) The Shenzhen, China dataset (CHN): The image format is PNG; the resolution of the images was variable but around 3000 × 3000 × 3 pixels, with 333 images of different TB patients and 329 images of normal controls (Figure 5a,b);

(2) The Montgomery dataset (MC): The image format is PNG; the resolution of the images was 4020 × 4892 × 3 or 4892 × 4020 × 3 pixels, with 58 images of different TB patients and 80 images of normal controls (Figure 5c,d).

For the sample set, we performed kernel density estimation (KDE) to map the empirical distribution of the overall datasets based on the finite sample, estimating the probability density function. The information of the datasets was fully used to avoid subjective prior knowledge; according to this function, we can determine the nature of the data distribution. The definition is shown in Equations (11) and (12):

(11) $$f\left(x\right)=\frac{1}{nh}\overset{n}{\underset{i=1}{\sum }}K_{0}\left(\frac{│x-x_{i}│}{h}\right)$$

(12) $$h=CxN^{-\frac{1}{5}}$$

In Equations (11) and (12), *K*_0_(*t*) is the kernel function. We use a Gaussian kernel function, where *x_i_* represents the one-dimensional sample data; *h* represents bandwidth, and can be calculated by Equation (12); *C* is a constant, taken as 1.05; and *N* represents the standard deviation of the one-dimensional sample data.

**Figure 6:**
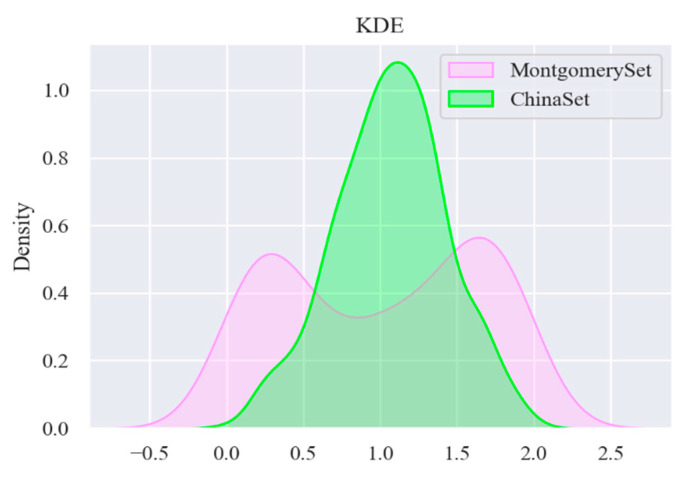
Visualization of KDE (kernel density estimation) distribution of two datasets.

The results of applying the KDE to the two datasets are shown in Figure 6. The shape of empirical distribution of the CHN dataset is single-peaked, while the empirical distribution of the MC dataset is double-peaked; therefore, through the empirical distribution, there is a big difference between the two datasets, and it is necessary to divide them.

In order to ensure that the training, validation, and testing of the model could be finished in two differently distributed datasets, the MC and CHN image datasets were divided into 80% training and 20% testing subsets, and 20% of the training data were used for validation, as shown in Table 3.

It should be noted that, considering that the data enhancement technology (random cropping and scaling) may cause the lesion area at the edge of the chest X-ray image to be omitted, the model training only uses rotation operation (no more than 6 degrees to the left and right), horizontal flip, and vertical flip. At the same time, the sizes of the images in the dataset are scaled to 512 × 512 × 24 by linear interpolation, and 16 images are packed into a batch for the model training. The split ratio of the training set and test set is 4:1. The test set is not used for model training, and serves for the final testing.

### 3.3. Related Design Experiment

#### 3.3.1. Distribution Radio of Basic Block

The basic block (Figure 3) is the main part of the model to extract features, and its number is closely related to the accuracy of model detection. As mentioned above, the output channels of the five basic blocks in the network structure are 16, 32, 48, 64, and 128, respectively. In order to find the optimal number of basic blocks, the number of basic blocks in different positions is appropriately adjusted on the basic network structure, and the parameters, FLOPS (floating-point operations), and accuracy of the network are tested experimentally. In Table 4, the column Module Ratio represents the ratio of the number of inserted basic blocks, while the column FLOPS represents floating-point arithmetic, which can be understood as computational complexity, and can be used to measure the complexity of the algorithm/model. It can be seen from Table 4 that the parameters and FLOPS of the model increase with the increase in the number of basic blocks. We argue that the network simply accumulating basic blocks cannot effectively improve the accuracy of the model. As can be seen from the first and third rows of Table 4, the third row’s model parameters increased by 94.4%, while the accuracy increased by only 0.6%, so the heavy structure may increase the burden of the model. We weighed the parameters and accuracy of the model and, finally, we used 1:1:1:1:1 to construct the network structure.

#### 3.3.2. Insertion Position of Channel Attention

Next, we investigated the ECA block (in Figure 3), which only involves a handful of parameters while bringing clear performance gain; its insertion position and number are closely related to model performance. Therefore, the aim of the experiment was to connect the ECA block after different basic blocks in order to explore the changes in the model’s accuracy.

In Table 5, the column Location represents the insertion position of the ECA block. Table 5 shows that the closer the ECA block is to the fully connected layer, the higher the accuracy of the model. When the number of the ECA blocks was increased, the accuracy decreased. We argue that the high-level semantic features extracted by deeper convolution layers are closely related to the network classification ability. After the high-level semantic features pass the information weighting of the ECA block(s), the key information in decision making is strengthened, while the useless information such as background is weakened, so the accuracy of model detection is increased. Therefore, this paper chooses to add the ECA block to the last basic block in order to achieve the highest prediction accuracy.

#### 3.3.3. Results of the Training Iteration Experiment

**Figure 7:**
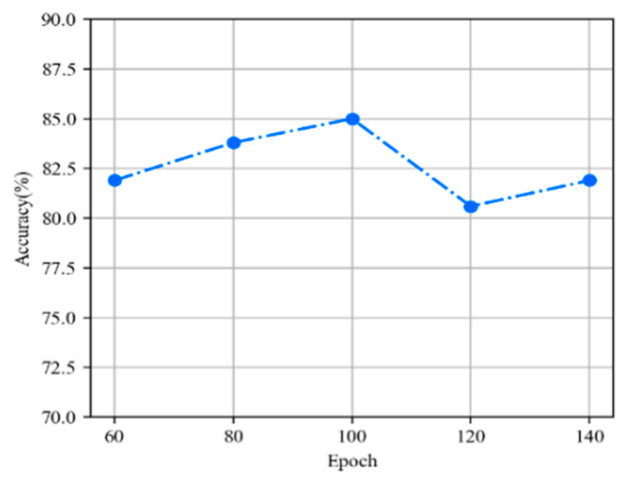
The changes in model accuracy at training iterations.

We explored the relationship between model accuracy and training iterations. Figure 7 shows that as training iterations increase, the deeper the model refinement, the more accurate the extraction of important features, so the accuracy of the model gradually increases. Remarkably, when the epoch is 120, the accuracy of the model drops by 4.9%; this accuracy dip is not significant, because the model proposed in this article has a lightweight structure, fewer parameters, and a faster learning and reasoning process. If the number of training iterations is too high, the model will overfit the data and cause the generalization ability on the test set to be low. For the best results, we set the epoch to 100.

### 3.4. Comparative Experiments

#### 3.4.1. Overall Performance of E-TBNet

Through the above experiments, the model structure of E-TBNet was completely constructed; at the end of this part, we compared our E-TBNet with lightweight models, including SqueezeNet [18], ShuffleNet [19], and MobileNet [20,21]. The SqueezeNet proposed by Landola et al. achieves AlexNet-level accuracy on ImageNet with 50× fewer parameters, and can be compressed to less than 0.5 Mb. Zhang et al. [19] proposed ShuffleNet, which is specially designed for mobile devices with very limited computing power. Channel shuffling and point–group convolution were used to reduce the amount of network computation. MobileNet proposes a deep separable convolution, which shows great potential for decomposing networks.

**Figure 8:**
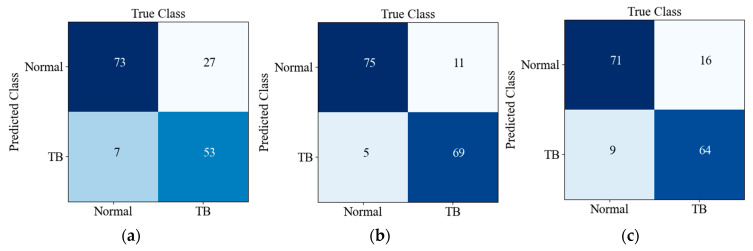

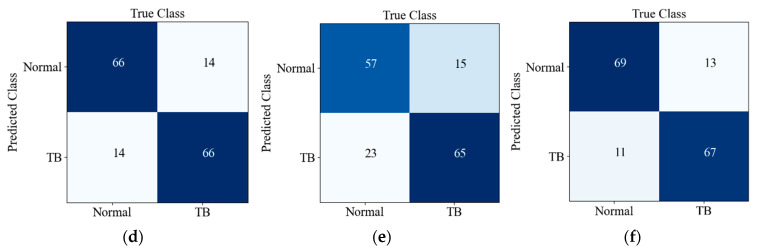
Confusion matrices of CNNs in this paper on the testing set: (**a**) MobileNet_v3_small; (**b**) MobileNetV2; (**c**) Shufflenet_V2_x0_5; (**d**) ShuffleNet_V2_x1_0; (**e**) SqueezeNet1_1; (**f**) E-TBNet.

Table 6 and Table 7 show the performance of different lightweight networks on the testing set, the results of which can be obtained through the confusion matrix in Figure 8, and all networks in this paper except for our network were initialized with pre-training weights.

It is not difficult to see from Table 6 that a network with pre-trained weights can achieve a higher accuracy. MobileNetV2 [20] achieves a maximum accuracy of 90%, because it has been fully trained on super-large-scale natural pictures for a long time, and it has a competitive feature extractor. Compared with our network, although the accuracy is reduced by 5%, the amount of model parameters is reduced by 77.7%, the computational complexity is reduced by 58.1%, and the weight of the model is compressed to only ~2 Mb, resulting in a faster reasoning efficiency.

The specificity of the MobileNet_v3_small [21] network reaches 91.2%, with good recognition ability for normal chest X-ray images; however, the recall rate of only 66.3% indicates that the model’s ability to recognize TB patients needs to be improved. Compared with other networks in the experiment, ShuffleNet_v2_x0_5 [19] has the fewest parameters and the smallest model weight, and it has comparative advantages in deploying and running on embedded devices, but the accuracy and recall rates are lower than those of our network. In disease detection, in order to reduce the missed diagnosis rate of the disease, it is necessary for the model to have higher recall and accuracy rates.

Table 6 and Table 7 show that the MobileNetV2 and MobileNet_v3_small tend to predict the sample as normal, so they have higher specificity, PPV, +LR, and –LR, but a lower recall rate. Combining recall and precision rate for analysis, MobileNet_v3_small’s F1-score is lower. The PPV/precision, +LR, and F1-score of ShuffleNet_v2_x1_0 and SqueezeNet1_1 are lower, so when the prediction result is positive, the probability of the sample being a true positive is lower. From the perspective of clinical comprehensive indicators, MobileNetV2 has a slight advantage, but the research goal of this article is to achieve an efficient reasoning process of the network. Compared to other networks in this paper, mobileNetV2 is not a lightweight network.

Without pre-training weights, whether comparing from a single indicator or comprehensive indicators, our network’s performance is better and more efficient. At the same time, the amount of model parameters and computational complexity can be reduced, and it has considerable advantages for deployment on embedded devices with low levels of hardware, which is in line with the original intention of the network’s design.

Figure 8 shows the confusion matrix of the prediction results of six CNNs on the testing set. There are 160 images in the testing set, and the ratio of TB to Normal is 1:1; TB and Normal refer to positive and negative, respectively. Predicted Class and True Class respectively represent the predicted class and true label of the input image. It can be seen from Figure 8f that 13 out of 80 TB images were misclassified as normal, while 11 out of 80 normal X-ray images were misclassified as TB images. The detection accuracy and recall rate of our network are better; although the PPV/precision of our model is not superior, it balances these two indicators well, so it achieves a better F1-score (harmonic average of precision and recall).

#### 3.4.2. Embedded Platform Experiment

**Figure 9:**
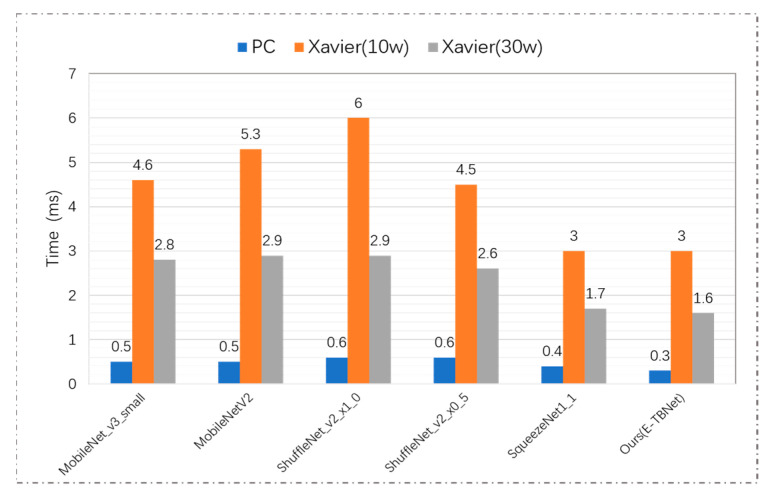
Comparison of the reasoning time of the CNNs used in this paper.

In order to compare the actual reasoning time of the above networks on an embedded device with limited computing power and storage resources, the lightweight CNNs used in this paper were deployed on the NVIDIA Jetson AGX Xavier platform and the PC, and the reasoning time for a single image under different hardware environments (PC, Xavier (10 W) and Xavier (30 W)) was recorded; the results are shown in Figure 9. Under the power of 10 W and 30 W, the reasoning time of TBNet is 3.0 ms and 1.6 ms, respectively. Compared with MobileNetV2, the E-TBNet in both modes can reduce the time consumption by ~44%. Although the performance of Shufflenet_V2_x0_5 and our network on the testing set is similar, our network has better reasoning efficiency.

In general, the heavy MobileNetV2 has the best recognition effect, but it is not suitable for fast-predicting embedded devices and PCs with low levels of hardware. In contrast, other lightweight models can achieve rapid prediction on the PC and Jetson AGX Xavier, but there is still a gap in the recognition accuracy. E-TBNet has the advantages of the above two aspects of classification networks; it can not only be easily deployed on devices with limited hardware conditions, but also accurately identify chest X-ray images of tuberculosis patients.

#### 3.4.3. The Results of Machine Learning Algorithms

**Figure 10:**
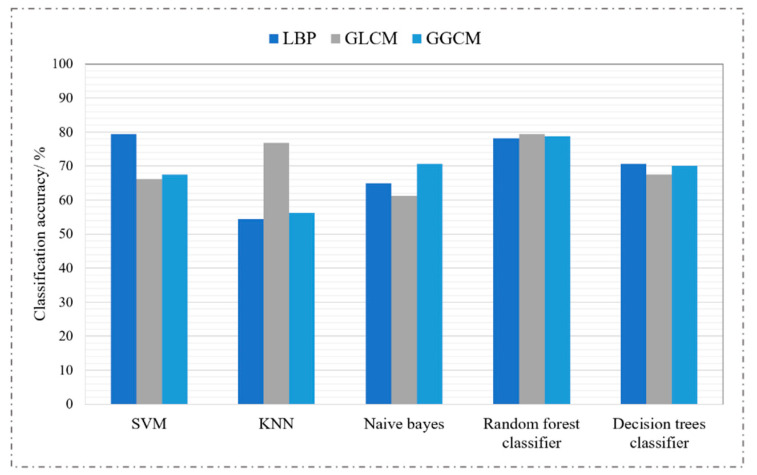
The results of five classification algorithms under different characteristics.

First, we used three feature extraction algorithms—local binary pattern (LBP) [22], gray-level co-occurrence matrix (GLCM) [23,24], and gray-level gradient co-occurrence matrix (GGCM) [25]—to extract texture features and grayscale features of chest radiographs. Then, we used five classic machine learning algorithms for classification: support-vector machine (SVM), *k*-nearest neighbors (KNN), naive Bayes, a random forests classifier, and a decision trees classifier. Finally, we evaluated the performance of the classifiers on the testing set. The grid optimization method was used to find the optimal parameters of the classifiers, and the experimental results are shown in Figure 10.

It is not difficult to see from Figure 10 that traditional machine learning algorithms have low accuracy for tuberculosis recognition tasks. Even if the same classifier is used to classify the features extracted by different feature extractors, the accuracy of the recognition is very different. Feature extraction is the key to machine learning tasks; in order to achieve high accuracy of model recognition, it is necessary for the algorithm designer to have a wealth of professional knowledge in order to manually extract high-quality distinguished tuberculosis lesion features. As is well known, the convolution kernel in deep learning has stronger feature-extraction capabilities, but its only disadvantage is that the reasoning time is longer than machine learning.

## 4. Discussion

When deep learning technology is applied in clinical practice, it will face greater challenges. We propose an efficient tuberculosis identification network that does not require large-scale data and has a faster reasoning process, but also has some limitations; for example, (1) its input must be 512 × 512 × 3 PNG images, and it cannot adapt to the image size; (2) the images collected by different DR devices have differences in brightness, grayscale, etc., which will make the network unstable; and (3) The Digital Imaging and Communications in Medicine (DICOM) data generated by the DR equipment must be converted into PNG images before they can be used for model classification.

## 5. Conclusions

The classification neural network has great application value in the early screening of TB in primary hospitals. This paper proposed a lightweight TB recognition network for PCs and Jetson AGX Xavier devices with lower hardware levels, then deployed it locally. In order to ensure that the network fully trains, validates, and tests on the data of different distributions, the two datasets were divided and fused, and the improved residual module and efficient channel attention were introduced to form the lightweight tuberculosis recognition model E-TBNet. The comparative experiment proved that compared with the optimal MobileNetV3 network for the PC, the network proposed in this paper sacrifices 4.8% accuracy, the number of parameters is reduced by 77.7%, the computational complexity is reduced by 58.1%, and the calculation speed of the model is effectively improved. In deep neural networks, the sample quantity and quality of the dataset determine the accuracy of the network model. Due to the lack of high-quality datasets in some primary hospitals, a lightweight network that uses a small number of samples to satisfy training will have greater clinical significance. However, the actual application environment is complex and changeable, and the generalization and robustness of the model need further research. In the future, based on the present work, the network could be designed to be more efficient and lightweight, while reducing its dependence on the hardware level and further improving the recognition accuracy of the network.

## Figures and Tables

**Table 1:** Hyperparameters for model training.

Hyperparameter	Value
Optimizer	SGD
Loss function	Cross-entropy
Batch size	16
Initial learning rate	0.001

**Table 2:** Two different hardware environments for model inference (PC and Jetson Xavier).

	Jetson Xavier (2018)	Personal Computer
GPU	Volta	Titan V
CPU	8 core ARM	6 core i5-9600KF
Memory	16 GB LPDDR4X	16 GB DDR4
Bandwidth	137 GB/s	652 GB/s
CUDA cores	512	5120

**Table 3:** Fusion of two differently distributed datasets (MC and CHN datasets).

Database	Types	Train Set/Fold	Test Set/Fold	All
Montgomery	Tuberculosis	46	12	58
	Normal	65	15	80
Shenzhen	Tuberculosis	268	65	333
	Normal	261	68	329

**Table 4:** The results of the ablation experiment of the distribution ratio of basic blocks.

Module Ratio	Parameters	FLOPS	Accuracy
1:1:1:1:1	0.486 × 10^6^	0.126 × 10^9^	85%
2:2:2:1:1	0.554 × 10^6^	0.163 × 10^9^	82.5%
2:2:2:2:2	0.945 × 10^6^	0.167 × 10^9^	85.6%

**Table 5:** Comparison of parameters, accuracy, and FLOPS of the ECA block in the insertion position ablation experiment.

Location	Parameters	FLOPS	Accuracy
1,2,3,4,5	0.486 × 10^6^	0.126 × 10^9^	80.1%
5	0.486 × 10^6^	0.126 × 10^9^	85.0%
4	0.486 × 10^6^	0.126 × 10^9^	83.1%
3	0.486 × 10^6^	0.126 × 10^9^	82.5%
2	0.486 × 10^6^	0.126 × 10^9^	82.5%
1	0.486 × 10^6^	0.126 × 10^9^	83.1%

**Table 6:** Comparison of multiple indicators of lightweight networks on the tuberculosis test dataset.

Model	Parameters	Sensitivity	Specificity	Accuracy	FLOPs	Weight
MobileNet_v3_small	1.52 × 10^6^	66.2%	91.2%	78.7%	0.6 × 10^9^	6.1 Mb
MobileNetV2	2.2 × 10^6^	86.2%	93.7%	90.0%	3.1 × 10^9^	8.9 Mb
ShuffleNet_v2_x0_5	0.34 × 10^6^	80.0%	88.7%	84.3%	0.4 × 10^9^	1.5 Mb
ShuffleNet_v2_x1_0	1.2 × 10^6^	82.5%	82.5%	82.5%	1.5 × 10^9^	5.1 Mb
SqueezeNet1_1	0.72 × 10^6^	81.2%	71.2%	76.2%	2.7 × 10^9^	2.8 Mb
Ours(E-TBNet)	0.49 × 10^6^	83.8%	86.3%	85.0%	1.3 × 10^9^	1.9 Mb

**Table 7:** Comparison of clinical comprehensive indicators of lightweight networks on the tuberculosis test dataset.

Model	+LR	−LR	F1-Score	PPV/Precision	NPV	Time
MobileNet_v3_small	7.57	0.37	0.757	0.883	0.730	0.5 ms
MobileNetV2	13.8	0.14	0.896	0.932	0.872	0.5 ms
ShuffleNet_v2_x0_5	7.11	0.22	0.836	0.876	0.816	0.6 ms
ShuffleNet_v2_x1_0	4.71	0.21	0.825	0.825	0.825	0.6 ms
SqueezeNet1_1	2.82	0.26	0.773	0.738	0.791	0.4 ms
Ours (E-TBNet)	6.09	0.19	0.848	0.860	0.841	0.3 ms

## Data Availability

Data sharing not applicable.

## Abbreviations

DR	Digital radiography
WHO	World Health Organization
CNN	Convolutional neural network
TB	Tuberculosis
AUC	Area under the curve
UM	Unsharp masking
CTD	Computer-aided tuberculosis diagnosis
TBNN	Tuberculosis neural net
ECA	Efficient channel attention
ResNet	Deep residual network
SENet	Squeeze-and-excitation network
*TP*	True positive
*TN*	True negative
*FP*	False positive
*FN*	False negative
PC	Personal computer
NLM	National Library of Medicine
CHN	China dataset
MC	Montgomery dataset
+*LR*	Positive likelihood ratio
−*LR*	Negative likelihood ratio
*PPV*	Positive predictive value
*NPV*	Negative predictive value
KDE	Kernel density estimation
FLOPS	Floating-point operations
TFLOPS	Trillion floating-point operations per second
SVM	Support-vector machine
KNN	*k*-Nearest Neighbors
GLCM	Gray-level co-occurrence matrix
GGCM	Gray-level gradient co-occurrence matrix
LBP	Local binary pattern
DICOM	Digital Imaging and Communications in Medicine
